# The Association of Retinal Disease with Vision Impairment and Functional Status in Medicare Patients

**DOI:** 10.36469/001c.93022

**Published:** 2024-03-29

**Authors:** Vincent Garmo, Xiaohui Zhao, Carmen D. Ng, Aimee Near, Tania Banerji, Keiko Wada, Gary Oderda, Diana Brixner, Joseph Biskupiak, Ferhina S. Ali, Archad M. Khanani, Alicia Menezes, Ibrahim M. Abbass

**Affiliations:** 1 Genentech, Inc., South San Francisco, California, USA; 2 IQVIA, Durham, North Carolina, USA; 3 University of Utah, Salt Lake City, Utah, USA; 4 New York Medical College, Valhalla, New York, USA; 5 Sierra Eye Associates, Reno, Nevada; 6 School of Medicine University of Nevada, Reno

**Keywords:** neovascular age-related macular degeneration, diabetic macular edema, retinal vein occlusion, quality of life, Medicare Current Beneficiary Survey

## Abstract

**Background:** The association of neovascular age-related macular degeneration (nAMD), diabetic macular edema (DME), and retinal vein occlusion (RVO) with functional status in the general Medicare population are not well established.

**Objectives:** This study examined patient-reported survey data linked with Medicare claims to describe the burden of these vision-threatening retinal diseases (VTRDs) among Medicare beneficiaries.

**Methods:** Medicare Current Beneficiary Survey data linked with Medicare Fee-for-Service claims data from 2006 to 2018 were used in a nationally representative retrospective pooled cross-sectional population-based comparison study. Outcomes between community-dwelling beneficiaries with nAMD (n = 1228), DME (n = 101), or RVO (n = 251) were compared with community-dwelling beneficiaries without any VTRDs (n = 104 088), controlling for baseline demographic and clinical differences. Beneficiaries with a diagnosis of nAMD, DME, or RVO during the data year were included; those with other VTRDs were excluded. Outcomes included vision function and loss, overall functioning as assessed by difficulties with activities of daily living (ADLs) and instrumental ADLs (iADLs), anxiety/depression, falls, and fractures. Results: In patient cohorts with nAMD, DME, and RVO, approximately one-third (34.2%-38.3%) reported “a little trouble seeing” (vs 28.3% for controls), and 26%, 17%, and 9%, respectively, reported “a lot of trouble seeing/blindness” (vs 5% of controls). Difficulty walking and doing heavy housework were the most reported ADLs and iADLs, respectively. Compared with those without VTRDs, beneficiaries with nAMD had higher odds of diagnosed vision loss (odds ratio [OR], 5.39; 95% confidence interval, 4.06-7.16; P < .001) and difficulties with iADLs (odds ratio, 1.41; 95% confidence interval, 1.11-1.80; P = .005); no differences were observed for DME or RVO vs control. After adjusting for age, sex, race/ethnicity, poverty status, comorbidities, and other relevant covariates, nAMD, DME, and RVO were not significantly associated with anxiety/depression, falls, or fractures.

**Discussion:** Patients with nAMD or DME were more likely to report severe visual impairment than those without VTRDs, although only those with nAMD were more likely to be diagnosed with vision loss.

**Conclusions:** Patients with nAMD continue to experience more vision impairment and worse functional status compared with a similar population of Medicare beneficiaries despite availability of therapies like antivascular endothelial growth factor to treat retinal disease.

## BACKGROUND

Although the etiologies of the chronic retinal vascular diseases neovascular age-related macular degeneration (nAMD), diabetic macular edema (DME), and retinal vein occlusion (RVO) differ, the vascular endothelial growth factor (VEGF) pathway is a pivotal component of the pathophysiology underlying all 3 conditions.^1^ Another common feature of these 3 disease states is that all can result in severe vision loss if left untreated.^2^

Previous research has linked having poor vision to an increased risk of falls and associated injuries, including fractures.^3–6^ Self-reported vision impairment also has been linked to an increased fear of falling, which can lead to activity limitation and associated declines in quality of life.^4^ Recently, psychological distress was reported in 26% of the adults who have difficulty seeing even when wearing glasses or contact lenses.^7^ In fact, patients with age-related macular degeneration (AMD) are at particularly high risk of depression compared with patients with other eye diseases.^8^ For community-dwelling older people, those with macular degeneration have twice the incidence of depression as those without, driven by both functional decline and loss of leisure activities.^9^

Vision loss has also been associated with disabilities in activities of daily living (ADLs) and instrumental activities of daily living (iADLs).^10^ Visually impaired individuals 60 years of age and over were recently shown to have significantly lower mean iADL scores compared with those without vision impairment, with iADL score being significantly correlated with near visual acuity.^11^ The relationship between retinal diseases and patient’s functional status relative to the Medicare population without vision-threatening retinal diseases (VTRDs) is not well described. Thus, this retrospective, real-world study sought to assess differences in perceived visual function, ADLs, iADLs, falls/fractures, and depression/anxiety between patients with nAMD, DME, or RVO and those without these or other common VTRDs, using data derived from the Medicare Current Beneficiary Survey (MCBS) linked with Medicare Fee-for-Service (FFS) claims.

## METHODS

### Study Design

This study analyzed pooled cross-sectional MCBS data linked with Medicare FFS claims from 2006 to 2013 and 2015 to 2018. The year 2014 was not included since 2014 data were not released by the Centers for Medicare & Medicaid Services.^12^ The MCBS is a continuous in-person longitudinal survey that collects individual-level data on beneficiaries’ largely self-reported sociodemographics, health status and functioning, access to care, health insurance coverage and expenses, financial resources, and family support. By design, the MCBS cross-sectional data are representative of the population of all Medicare beneficiaries for any given survey year.^13^ This study was exempt from Institutional Review Board approval due to de-identified data.

### Study Population

The study population consisted of community-dwelling (ie, not living in a nursing home or other facility) adult (≥18 years) Medicare beneficiaries who had full-year Medicare Parts A and B enrollment in the same year of administration of the MCBS survey. The disease cohorts included beneficiaries who were diagnosed with nAMD, DME, or RVO. To increase diagnostic specificity, beneficiaries with at least 1 inpatient diagnosis or at least 2 outpatient diagnoses of nAMD, DME, or RVO during the data year were included.^14^ Diagnoses were identified from the Medicare FFS claims based on the *International Classification of Diseases, Ninth or Tenth Revision, Clinical Modification* (ICD-9-CM or ICD-10-CM) diagnosis codes for nAMD, DME, and RVO (**Supplementary Excel File**).^15,16^ Patients were classified to mutually exclusive cohorts based on the first diagnosis observed in the data year (ie, nAMD, DME, and RVO cohorts). Patient diseases were not required to be incident, and the length of time a patient had the disease of interest was not captured by the study data. Patients were not required to be on active treatment. The comparator cohort consisted of Medicare beneficiaries without any VTRDs, including nAMD, DME, and RVO.

Other inclusion criteria included survival through the data year. Individuals with VTRDs other than the conditions of interest or more than 1 VTRD were not included in this study. Examples of exclusionary diseases were endophthalmitis, central artery occlusion, severe glaucoma, surgery for glaucoma (eg, tube-shunt, trabeculectomy), proliferative diabetic retinopathy, congenital cataract, optic atrophy, amblyopia, optic neuritis, neuromyelitis optica, and ischemic optic neuropathy.

### Outcomes

Outcomes were assessed from both MCBS questionnaires and Medicare claims data. ADLs, iADLs, and perceived visual function were collected from the MCBS responses. Difficulties with ADLs and iADLs were defined as stage 0 (no difficulties), stage I (mild), stage II (moderate), stage III (severe), and stage IV (complete) (**Supplementary Table S1**).^17^ Additionally, diagnosis of vision loss and anxiety were identified from Medicare claims based on ICD-9-CM or ICD-10-CM codes. Evidence of depression, falls, and fractures were identified by either diagnosis codes from Medicare claims or responses to corresponding MCBS queries.

Relationships between the outcomes of interest and baseline clinical and demographic characteristics were investigated. The characteristics examined included socioeconomic characteristics and overall health status derived from the MCBS responses, as well as Charlson Comorbidity Index (CCI) scores determined from Medicare claims with Quan’s adaptation (**Supplementary Table S2**).^18^

### Statistical Analysis

The outcomes were compared between each disease cohort and the control cohort. For bivariate analyses, parametric F-test (mean) and nonparametric Wilcoxon rank-sum test (median) were used to compare continuous variables (eg, age, CCI scores) between 2 groups. Rao-Scott chi-square tests were used to compare binary variables (eg, presence of falls, fractures, and depression/anxiety) and categorical (eg, ADL/iADL status) variables among 2 or more groups.

Adjusted models were developed only when bivariate relationships were statistically significant. Logistic regression was used to assess differences in perceived visual function, diagnosed vision loss, and presence of falls, fractures, and depression/anxiety (ie, evidence of either depression or anxiety) between those with retinal diseases of interest and the control cohort. The association of the retinal disease of interest with ADL/iADL status was explored with multinomial logistic regression. For each such analysis, the covariate list was determined upon results of bivariate analyses that compared the corresponding disease cohort with the control cohort and expert opinions derived from clinical input and published literature. All models were adjusted for age, sex, race/ethnicity, and poverty status of Medicare beneficiaries. In the visual function model, additional covariates included the presence of cataract(s) and glaucoma; the additional covariates for the ADL/iADL status models included CCI categories, the presence of arthritis, dementia, hypertension, and osteoporosis.

Balanced repeated replication weights were used for variance estimation in the bivariate tests and regression models to account for nonindependence of the person-years in the multiple year pooled dataset, yielding pooled estimates that represent a moving average of nationally representative year-specific estimates. MCBS weights accounted for potential nonresponse and sample coverage bias. Annualized weighted Ns and percentages were reported to represent the Medicare population. The pooled estimates can be interpreted as being representative of the midpoint of the pooled period (ie, year 2012). The annualized weighted Ns were derived by dividing weighted Ns by the total number of data years (ie, 12) to represent the average number of Medicare beneficiaries in a given year.

A *P* value <.05 was considered statistically significant for all analyses. All analyses accounted for the complex survey design by using survey procedures with Statistical Analysis System version 9.4 software (SAS Institute Inc.).

## RESULTS

### Study Sample

**Figure 1.**
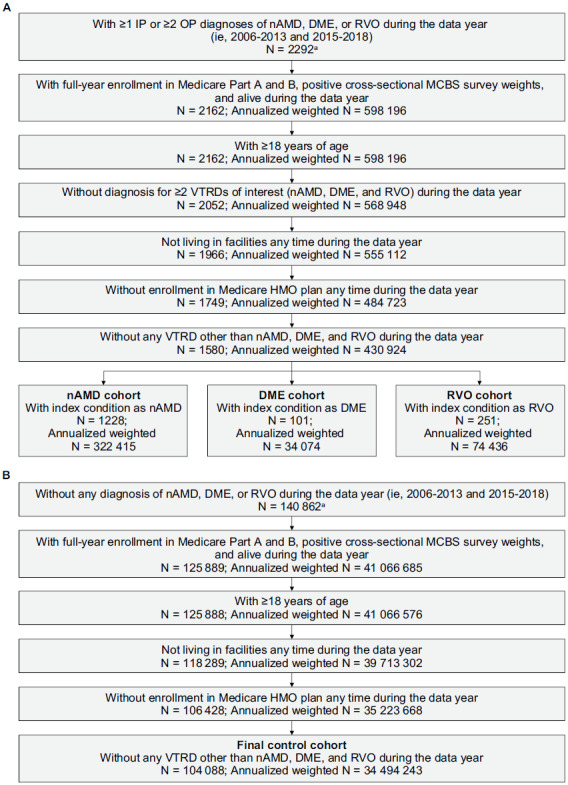
Selection of the nAMD, DME, and RVO **(A)** and Control Cohorts **(B)** Annualized weighted N was derived by dividing the weighted N by the total number of data years (ie, 12). ^a^Not weighted since generated from claims data rather than survey data. Abbreviations: DME, diabetic macular edema; HMO, health maintenance organization; IP, inpatient; MCBS, Medicare Current Beneficiary Survey; nAMD, neovascular age-related macular degeneration; OP, outpatient; RVO, retinal vein occlusion; VTRD, vision-threatening retinal disease.

A total of 2292 patients, representing more than 7 million of the weighted population, with nAMD, DME, or RVO during the data years of 2006 to 2013 and 2015 to 2018 were identified. Of these, 1580 (annualized weighted N = 430 924) fulfilled the eligibility criteria for the overall study cohort, including 1228 (annualized weighted N = 322 415) patients with nAMD, 101 (annualized weighted N = 34 074) with DME, and 251 (annualized weighted N = 74 436) with RVO (**Figure 1A**). For the control cohort, 104 088 (annualized weighted N = 34 494 243) beneficiaries fulfilled eligibility criteria (**Figure 1B**).

### Unadjusted Demographic and Socioeconomic Characteristics

Patient characteristics differed between the nAMD, DME, and RVO cohorts and the control group (**Table 1**). The nAMD cohort was the oldest among the 4 cohorts studied (age in mean years [SE]: nAMD, 83.0 [0.4]; DME, 71.6 [1.5]; RVO, 78.8 [0.7]; control, 72.0 [0.1]), and the nAMD and RVO cohorts were both significantly older than the control cohort (*P <* .0001). Compared with the control cohort, the nAMD cohort had a significantly higher proportion of females (61.6% vs 55.7%, *P =* .0035), non-Hispanic White people (95.6% vs 83.0%, *P <* .0001), nonmetropolitan residents (28.3% vs 23.2%, *P =* .0031), and beneficiaries with income greater than 200% above the federal poverty line (60.1% vs 55.1%, *P =* .0029). The nAMD and DME cohorts included a significantly higher proportion of beneficiaries receiving veteran benefits than the control cohort (control: 5.2%; vs nAMD: 8.9%, *P* = .0001; vs DME: 16.6%, *P <* .0001). Use of anti-VEGF injections was significantly higher (*P <* .0001) in all 3 cohorts (n [%]: nAMD, 919 [74.6]; DME, 46 [48.0]; and RVO, 109 [41.9]) than in the control cohort (1504 [1.5]).

**Table 1.** Unadjusted Demographic and Socioeconomic Characteristics of Study Cohorts During the Data Year

**Cross-sectional Characteristics During the Data Year**	**Control (N = 104 088; Annualized Weighted N = 34 494 243), Weighted %**	**nAMD (N = 1228; Annualized Weighted** **N = 322 415)**		**DME (N = 101; Annualized Weighted** **N = 34 074)**		**RVO (N = 251; Annualized Weighted N = 74 436)**	
		**Weighted %**	***P* Value^a^**	**Weighted %**	***P* Value^a^**	**Weighted %**	***P* Value^a^**
**Demographic characteristics**							
Age on index date (years)							
Mean (SE)	72.0 (0.1)	83.0 (0.4)	<.0001	71.6 (1.5)	.7649	78.8 (0.7)	<.0001
Median (Q1-Q3)	72.1 (67.0-⁠78.9)	83.1 (78.4-⁠87.2)		72.5 (68.4-⁠76.4)		79.0 (72.9-⁠84.0)	
Age categories at index							
18-74 years	57.8^b^	11.0^c^	<.0001	60.8^d^	.4627	30.7^e^	<.0001
75-84 years	30.5	45.2		32.1		44.5	
≥85 years	11.7	43.9		7.1^f^		24.8	
Sex							
Male	44.3	38.4	.0035	49.8	.4022	41.3	.4585
Female	55.7	61.6		50.2		58.7	
Race/ethnicity							
Non-Hispanic White	83.0	95.6	<.0001	80.5	.6435	90.0	.0772
Others	17.0	4.4		19.5		10.0	
US Census Bureau geographic region							
Northeast	18.7	20.9	.2273	28.0	.0383	19.8	.5476
Midwest	22.3	24.4		30.8		21.5	
South	38.2	36.1		26.4		42.8	
West and other	20.9	18.6		14.8		15.9	
Metropolitan status							
Metropolitan area	76.8	71.7	.0031	65.9	.0719	74.6	.6295
Other	23.2	28.3		34.1		25.4	
Index year							
2006-2010	37.4	37.0	.2757	24.1	.0079	34.2	.3635
2011-2015	33.3	31.0		50.0		31.8	
2016-2018	29.4	32.0		26.0		34.1	
**Socioeconomic characteristics**							
Highest education level^g^							
Less than high school	22.4	18.7	.1046	32.5	.1955	18.8	.7260
High school or equivalent	36.4	40.2		33.3		36.4	
More than high school	40.8	40.5		34.0		44.4	
Marital status							
Married	49.9	42.6	<.0001	56.4	.5759	51.1	.0003
Widowed	25.8	46.3		19.6		36.6	
Other	24.3	11.2		24.1		12.3	
Poverty status^h^							
≤100% of FPL	16.6	11.8	.0029	13.3	.6579	11.1	.1334
>100% and ≤200% FPL	28.3	28.1		32.0		25.9	
>200% FPL	55.1	60.1		54.8		63.0	
Any Part D coverage	73.8	60.0	<.0001	69.0	.3663	63.4	.0013
Other payers							
Any Medicaid	18.2	9.6	<.0001	22.9	4027	12.4	.1476
Any private insurance	47.8	82.0	<.0001	60.3	.0670	77.2	<.0001
Any veteran benefits	5.2	8.9	.0001	16.6	<.0001	8.5	.0677

Annualized weighted N was derived by dividing the weighted N by the total number of data years (ie, 12).

^a^*P* value was based on comparison between the control cohort and the nAMD/DME/RVO cohort.

^b^16.0% (weighted %) of control were under 65 years of age.

^c^1.2% (weighted %) of nAMD were under 65 years of age.

^d^12.8% (weighted %) of DME were under 65 years of age.

^e^1.9% (weighted %) of RVO were under 65 years of age.

^f^Results with relative standard error >30% and may be considered not reliable due to low cell sizes.

^g^<1% of patients in each cohort had missing data on educational level.

^h^Poverty status was determined based on responses to inquiry about individual income and the federal poverty line of the index year.

Abbreviations: DME, diabetic macular edema; FPL, federal poverty level; nAMD, neovascular age-related macular degeneration; RVO, retinal vein occlusion; SE, standard error of the mean.

The burden of comorbidities was significantly higher among the nAMD, DME, and RVO cohorts compared with the control cohort (see **Table 2**). Rates of CCI scores of 3 or more for the nAMD, DME, and RVO groups were 26.4%, 54.3%, and 32.5%, respectively, compared with 12.8% for the control cohort. Most ocular comorbidities and many nonocular comorbidities occurred at a higher frequency in the nAMD, DME, and RVO cohorts compared with controls. The most prevalent ocular comorbidity across the retinal disease cohorts was cataracts, which were significantly more common in the nAMD, DME, and RVO cohorts (*P <* .0001; 60.6%, 68.5%, and 63.7%, respectively) than the control cohort (40.0%). Hypertension and cardiovascular disease were more prevalent in the nAMD, DME, and RVO cohorts than in the control cohort (hypertension [control 58.5% vs nAMD: 64.7%, *P =* .0011; vs DME: 76.5%, *P =* .0072; vs RVO: 70.2, *P =* .0101]; cardiovascular disease [control 37.6% vs nAMD: 68.3%, *P <* .0001; vs DME: 76.0%, *P* < .0001; vs RVO: 65.6, *P <* .0001]).

**Table 2.** Unadjusted Clinical Characteristics of the nAMD, DME, and RVO Cohorts Compared With the Control Cohort

	**Control (N = 104 088; Annualized Weighted N = 34 494 243)**	**nAMD (N = 1228; Annualized Weighted N = 322 415)**		**DME (N = 101; Annualized Weighted N = 34 074)**		**RVO (N = 251; Annualized Weighted N =74 436)**	
	**Weighted %**	**Weighted %**	***P* value^a^**	**Weighted %**	***P* value^a^**	**Weighted %**	***P* value^a^**
**Cross-sectional characteristics during the data year**							
CCI^b^							
Mean (SE)	0.9 (0.01)	1.7 (0.09)	<.0001	3.6 (0.45)	<.0001	1.9 (0.15)	<.0001
Median (Q1-Q3)	0 (0-0.7)	0.6 (0-2.2)		2.3 (0.8-5.1)		1.9 (0.15)	
CCI category							
0	66.2	38.9	<.0001	14.3^c^	<.0001	39.1	<.0001
1	12.3	19.8		13.2^c^		17.9	
2	8.6	14.9		18.2		10.5	
≥3	12.8	26.4		54.3		32.5	
Use of intravitreal anti-VEGF injections	1.5	74.6	<.0001	48.0	<.0001	41.9	<.0001
**Other comorbidities of interest^d^**							
Ocular diseases							
Cataracts	40.0	60.6	<.0001	68.5	<.0001	63.7	<.0001
Diabetic retinopathy	2.2	3.4	.0512	64.0	<.0001	9.4	<.0001
Glaucoma	8.1	15.1	<.0001	21.6	<.0001	20.3	<.0001
Nonocular diseases							
Arthritis	61.4	71.2	<.0001	69.3	.1958	63.1	.6277
Cardiovascular disease	37.6	68.3	<.0001	76.0	<.0001	65.6	<.0001
Dementia	5.9	7.8	.0131	5.9	.9830	7.1	.4262
Diabetes	30.9	30.0	.6510	99.2	<.0001	35.2	.2300
Dyslipidemia	41.4	44.1	.1725	60.3	.0013	44.6	.4717
Hypertension	58.5	64.7	.0011	76.5	.0072	70.2	.0101
Osteoporosis	22.1	30.0	<.0001	21.6	.9120	31.9	.0054
Renal disease	6.0	12.4	<.0001	34.4	<.0001	18.1	<.0001

Annualized weighted N was derived by dividing the weighted N by the total number of data years (ie, 12).

Abbreviations: CCI, Charlson Comorbidity Index; DME, diabetic macular edema; ICD-9-CM, *International Classification of Diseases, Ninth Revision, Clinical Modification*; ICD-10-CM, *International Classification of Diseases, Tenth Revision, Clinical Modification;* nAMD, neovascular age-related macular degeneration; RVO, retinal vein occlusion; VEGF, vascular endothelial growth factor.

^a^*P* value was based on comparison between the control cohort and the nAMD/DME/RVO cohort.

^b^Quan’s adaptation.

^c^May not be reliable as estimate has a relative standardized error >30%.

^d^Identified based on ICD-9-CM or ICD-10-CM diagnosis codes in claims or self-reported diagnoses from survey responses.

### Vision Impairment and Functional Status

**Figure 2.**
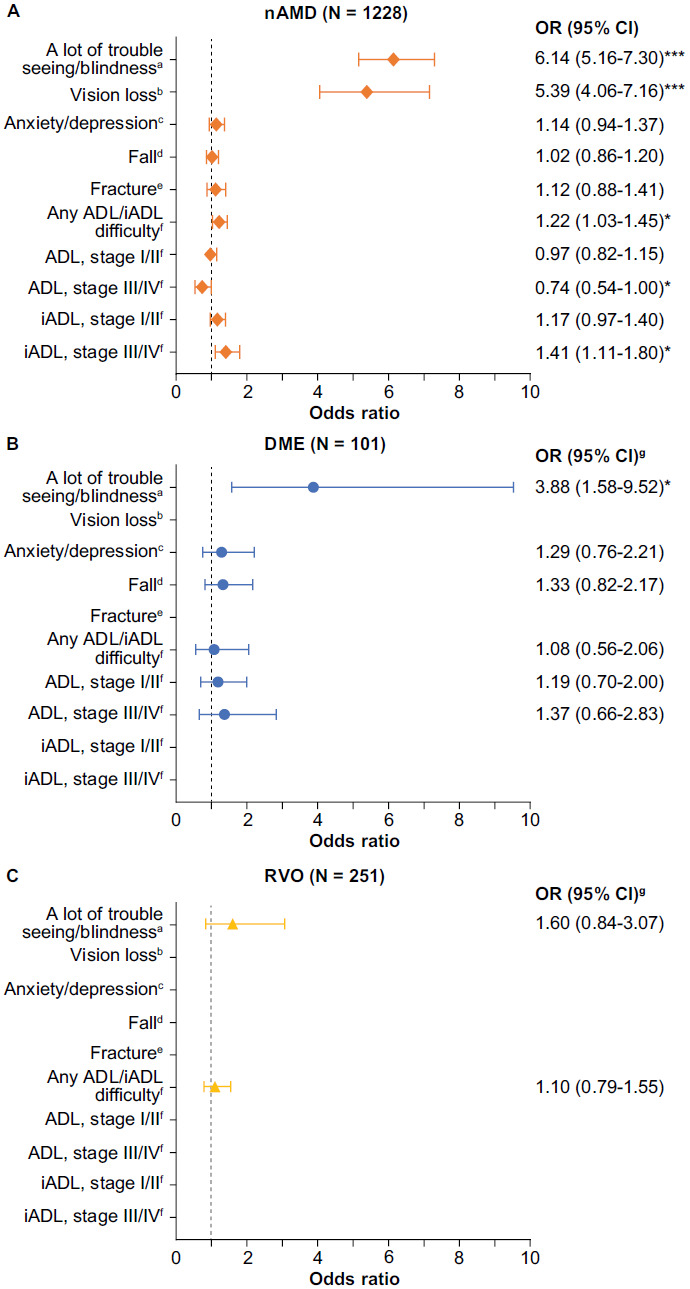
Association of Vision-Threatening Retinal Diseases with Vision and Overall Function Compared With Control Cohort ^a^Perceived visual function. The models are adjusted for age categories, sex, race/ethnicity, poverty status, the presence of cataract, and the presence of glaucoma. ^b^Diagnosed vision loss. The models are adjusted for age categories, sex, race/ethnicity, poverty status, the presence of cataract, and the presence of glaucoma. ^c^Models are adjusted for age categories, sex, race/ethnicity, poverty status, CCI categories, the presence of arthritis, dementia, hypertension, and osteoporosis (as well as diabetes for the model for DME vs control). ^d^Models are adjusted for age categories, sex, race/ethnicity, poverty status, CCI categories, the presence of cataract or glaucoma, arthritis, dementia, hypertension, and osteoporosis (as well as diabetes for the model for DME vs control). ^e^Model is adjusted for age categories, sex, race/ethnicity, poverty status, CCI categories, the presence of cataract or glaucoma, arthritis, dementia, hypertension, and osteoporosis. ^f^Stage 0 = not limited at all; stage I/II = mild to moderate limitation; stage III/IV = severe to complete limitation. The models for ADL and iADL status are adjusted for age categories, sex, race/ethnicity, poverty status, CCI categories, the presence of arthritis, dementia, hypertension, and osteoporosis (as well as diabetes for the model for DME vs control). ^g^OR was not calculated when bivariate analysis indicated no significant difference. *P< .05; **P < .001; ***P < .0001 (vs control cohort). Abbreviations: ADL, activities of daily living; CCI, Charlson Comorbidity Index; DME, diabetic macular edema; iADL, instrumental activities of daily living; CI, confidence interval; nAMD, neovascular age-related macular degeneration; OR, odds ratio; RVO, retinal vein occlusion.

When adjusted for covariates, patients with either nAMD (odds ratio [OR], 6.14; 95% confidence interval [CI], 5.16-7.30; *P <* .0001) or DME (OR, 3.88; 95% CI, 1.58-9.52; *P <* .01) were more likely to report “a lot of trouble seeing/blindness” compared with controls (**Figure 2**). Patients with nAMD, but not DME or RVO, had higher odds of having diagnosed vision loss compared with controls, even after adjusting for other characteristics, such as the presence of a cataract or glaucoma (OR, 5.39; 95% CI, 4.06-7.16; *P <* .001). Although depression and falls were the 2 most commonly reported comorbidities and complications in all cohorts, after adjusting for covariates, the retinal diseases of interest were not significantly associated with the presence of anxiety/depression, fall, or fracture (**Figure 2**).

Patients with nAMD, but not DME or RVO, were more likely to report difficulty with ADL/iADL (OR, 1.22; 95% CI, 1.03-1.45; *P <* .05) (**Figure 2**). Before adjusting for covariates, the most frequently reported difficulty with individual ADLs was walking in all VTRD and control cohorts (control: 27.1%; nAMD: 33.1%, *P* = .0025; DME: 39.1%, *P =* .0376; RVO: 27.6% *P* = .9430) (**Supplementary Excel File**). However, when adjusting for covariates, none of the cohorts of interest were associated with having a statistically significant difference in ADL difficulty, although borderline significance was seen for having stage III/IV difficulty when comparing nAMD vs control (OR, 0.74; 95% CI, 0.54-1.00; *P <* .05) (**Figure 2**). Before adjustment, doing heavy housework was the most frequently reported difficulty in iADL across all 4 cohorts (control: 34.6%; nAMD: 44.4%, *P* <.0001; DME: 44.0%, *P =* .1924; RVO: 38.7%, *P* = .5302) (**Supplementary Figure S1**). When adjusting for covariates, patients with nAMD had a higher likelihood of having stage III/IV iADL difficulty (OR, 1.41; 95% CI, 1.11-1.80; *P <* .05) but not stage I/II difficulty (OR, 1.17; 95% CI, 0.97-1.40; *P >* .05) (**Figure 2**). There was no difference in stage III/IV and stage I/II between DME or RVO cohorts and the control group.

## DISCUSSION

### Topline Summary

To our knowledge, this analysis is the first to assess patient-reported outcomes at the population level (using the MCBS linked with Medicare FFS claims) for nAMD, DME, and RVO. These analyses highlight the association between the disease and quality of life among community-dwelling Medicare beneficiaries with nAMD and DME. Patients with nAMD or DME were more likely to report severe visual impairment compared with those without VTRDs, although only those with nAMD were more likely to be diagnosed with vision loss. It is possible that vision loss was under-recorded via diagnosis codes. Given the selection criteria used in this study (eg, ≥1 inpatient or 2 outpatient diagnoses, full-year enrollment, and exclusion of other vision-threatening diseases), the annualized weighted N related to each study cohort was much smaller than the number of Medicare patients diagnosed with VTRDs in 2019 (nAMD: 547 744, DME: 284 235; branched RVO: 159 882; and central RVO: 94 745).^19^

### Impact on Quality of Life: ADLs, iADLS, and Complications/Comorbidities

Difficulty with heavy housework was the most commonly reported iADL for all cohorts. Unlike those in the DME and RVO cohorts, those in the nAMD cohort reported having more difficulties than those in the control cohort with using the telephone, shopping, managing money, and preparing meals. Loss of central vision can make it hard to use cell phones or read details on currency and packages, possibly explaining why these tasks could become more difficult for patients with nAMD. nAMD was associated with higher likelihood of reporting stage III/IV iADL difficulty, but not stage I/II difficulty. This may suggest the impact to a patients’ functional status may be relatively unaffected until central vision loss falls below a certain threshold, at which point the patient may experience severe loss of daily functional ability.

In previous research, patients with AMD were reported to experience more falls and injuries related to falls, with fear of falling contributing to a decrease in quality of life.^4–6^ In this investigation, about one-third of patients with nAMD, DME, or RVO in the Medicare population reported experiencing falls (33.3%, 41.4%, and 29.0%, respectively), compared with 27.7% of patients in the control cohort. However, after adjusting for age, sex, race/ethnicity, poverty status, comorbidities, and other relevant covariates, none of the retinal diseases of interest were associated with increased falls or fractures when compared with the control cohort. This may have been due to the relatively small number of patients to measure a relatively infrequent medical event or reflect real changes in outcomes arising from a combination of factors including better patient education, improved environments for seniors, and the increased use of anti-VEGF agents in the treatment of VTRDs since first approval in 2004 (shortly before the initial year from which our data were captured).^20^ Currently, intravitreal injections of anti-VEGF agents, generally given every 1 to 3 months, are the established standard-of-care treatment for these VTRDs and have been shown in clinical trials to be effective in improving or maintaining vision.^21–23^ With the use of these drugs in more recent years, the Medicare population may be experiencing less vision impairment overall than was the case at the time of the previous studies that did find an increase in falls and fractures; additional studies are needed to confirm this hypothesis.

In the past, patients reporting visual function loss have been shown to be at increased risk of depression.^24^ In this study, when compared with the control cohort, both anxiety and depression were more common in the nAMD cohort, and depression was more common in the DME cohort; however, neither retinal disease was associated with either anxiety or depression after adjusting for relevant covariates. This may be because of the mean age of patients being over 70 years for all cohorts in the current investigation since the association of depression and vision loss is stronger in younger working-age adults compared with those 65 years of age and older.^7^ In addition, in studies exploring the association of visual impairment and depression, depression was less commonly reported in studies designed to detect multiple disabilities (like the MCBS) than in those with the primary aim of detecting depression.^25^

### Limitations

One of the most notable limitations of this investigation is that patient visual acuity outcomes are unknown, as the MCBS data have no linkage to electronic health records. Instead, vision loss was identified by a general self-reported assessment of vision difficulties or a diagnosis code, which may be underutilized in the real-world setting and used inconsistently across practices. Additionally, the cross-sectional design of this study does not ensure that visual function measures, ADLs, and iADLs (typically collected near the end of the year) were collected after disease diagnosis (which could occur any time that year). Given the focus upon disease burden, we did not seek to determine any relationship between exposure (ie, management of disease) and outcome. Since this study utilized the MCBS survey responses that were reported by Medicare beneficiaries or their proxies, it is prone to the limitations commonly observed in other studies based on survey data (eg, recall bias). Another limitation is that the sample sizes for DME and RVO cohorts were relatively small compared with the nAMD cohort, which did make it more difficult to detect significant differences. Since this study included a sample of Medicare beneficiaries enrolled in the FFS plans, the study results may not be generalized to Medicare beneficiaries enrolled in managed care plans. Similarly, the results may not be generalizable to patients who changed their insurance plans since full-year enrollment in Medicare Parts A and B was required for inclusion in this study. Further analyses of the MCBS data linked with claims are warranted to explore the role of treatments for nAMD or DME in improving perceived visual function and overall functional status in the Medicare population.

### Implications

These findings suggest there is still significant disease burden in patients with VTRDs, especially nAMD. Patients with nAMD continue to experience more vision loss and reported a lot of trouble seeing and blindness significantly more often than patients in the control cohort even though most nAMD patients (75%) received anti-VEGF treatment. Potential reasons for this include poor responsiveness to existing treatment options seen in some patients with nAMD and high treatment burden due to the frequency of ongoing injections. A similar but nonsignificant trend was also observed in patients with DME and RVO. Solutions for improving vision and functional status in patients living with retinal diseases include facilitating greater patient education and care coordination to facilitate early screening of at-risk patients to prevent irreversible disease progression, as well as improving access to available treatments. Additionally, advanced interventions that improve effectiveness and reduce treatment burden may further reduce vision impairment associated with these retinal diseases.

## CONCLUSIONS

Patients with nAMD continue to experience more vision impairment and worse functional status compared with a similar population of Medicare beneficiaries despite availability of therapies like anti-VEGF agents to treat retinal disease. However, when compared with the control cohort, none of the retinal diseases of interest were associated with increased falls, fractures, or anxiety and depression after adjusting for relevant covariates.

### Disclaimer

The findings and conclusions in this report are those of the authors and do not necessarily represent the official position of the US Centers for Disease Control and Prevention.

### Disclosures

V.G., A.M., C.D.N., and I.M.A. are employees of Genentech, Inc. G.O., J.B., and D.B. have consultancies with Genentech, Inc. F.S.A. has consultancies with Allergan/AbbVie, Apellis, EyePoint, Genentech, Inc., and Regeneron and is on the speakers’ bureau of Apellis. A.M.K. has consultancies with 4D Molecular Therapeutics, AbbVie, Adverum, Aerie, Aldebaran, Allergan, Apellis, Applied Genetics Technologies Corporation, Arrowhead, Aviceda, Bausch + Lomb, Broadwing Bio, Clearside, Exegenesis Bio, EyePoint, Frontera, Genentech, Inc., Gyroscope, iLumen, Iveric Bio, Janssen, Kartos, Kato, Kodiak Sciences, Kriya, Nanoscope, Notal, Novartis, Ocular Therapeutix, Oculis, OcuTerra, Olives Bio, Opthea, Oxurion, Perfuse, PolyPhotonix, Protagonist, Ray Therapeutics, RecensMedical, Regeneron, Regenxbio, RevOpsis, Roche, Stealth, Thea, Unity, Vanotech, and Vial; has received funding and grants from 4D Molecular Therapeutics, Adverum, Annexon, Apellis, Genentech, Inc., Gyroscope, Iveric Bio, Kodiak, Neurotech, NGM Bio, Novartis, Ocular Therapeutix, Oculis, OcuTerra, Opthea, Oxurion, Regenxbio, Roche, and Unity; and reports stock ownership or options in RevOpsis. X.Z. and A.N. are employee of IQVIA. T.B. and K.W. were employees of IQVIA at the time of the study

### Presentation

Portions of the data were presented at The Professional Society for Pharmacoeconomics and Outcomes Research (ISPOR) and Academy of Managed Care Pharmacy (AMCP) Nexus Meetings in 2021.

## Supplementary Material

Supplementary Excel File

Online Supplementary Material
