# Supplementary material for: The Association of Retinal Disease with Vision Impairment and Functional Status in Medicare Patients
Source: J Health Econ Outcomes Res. 2024 Mar 29;11(1):94–102. doi: 10.36469/001c.93022 (PMC10981881; doi:10.36469/001c.93022)
Supplement: Online Supplementary Material [file jheor_2024_11_1_93022_221960.pdf]

Journal of Health Economics and Outcomes Research

Online Supplementary Material

The Association of Retinal Disease With Vision Impairment and Functional Status in Medicare Patients. *JHEOR*. 2024;11(1):94-102. doi:10.36469/jheor.2024.93022

Table S1. ADL and IADL Staging Definitions Based on MCBS Survey Responses

Table S2. Variable Source

Figure S1. Unadjusted Difficulties in Individual Activities

This supplementary material has been provided by the authors to give readers additional information about their work.

This is an open-access article distributed under the terms of the Creative Commons Attribution 4.0 International License (CCBY-4.0). View this license's legal deed at http://creativecommons.org/licenses/by/4.0 and legal code at http://creativecommons.org/licenses/by/4.0/legalcode for more information.

Table S1. ADL and iADL Staging Definitions Based on MCBS Survey Responses

**Limitation Expressed as Difficulty**	**ADL Stage**	**iADL Stage**
0 = None	Can eat, toilet, dress, bathe/shower, get in/out of bed/chairs, and walk without difficulty	Can use the telephone, manage money, prepare meals, do light housework, shop for personal items, and do heavy housework
I = Mild	Without difficulty in eating, toileting, dressing, and bathing/showering	Without difficulty in using the telephone, managing money, preparing meals, and doing light housework
II = Moderate	Without difficulty in eating and toileting	Without difficulty in using the telephone and managing money
III = Severe	With difficulty in eating or toileting but not with all ADLs	With difficulty in using the telephone or managing money but not all iADLs
IV = Complete	With difficulty in all ADLs	With difficulty in all iADLs

Reference: Stineman MG, Streim JE, Pan Q, Kurichi JE, Schussler-Fiorenza Rose SM, Xie D. Activity Limitation Stages empirically derived for activities of daily living (ADL) and instrumental ADL in the U.S. adult community-dwelling Medicare population. *PM R.* 2014;6(11):976-987. doi:10.1016/j.pmrj.2014.05.001
Abbreviations: ADL, activities of daily living; iADL, instrumental activities of daily living; MCBS, Medicare Current Beneficiary Survey.

Table S2. Variable Source

**Outcome**	**Data Source**
ADLs	MCBS survey responses
iADLs	MCBS survey responses
Perceived visual functions	MCBS survey responses
Vision loss	Medicare claims
Anxiety	Medicare claims
Depression	Medicare claims or MCBS survey responses
Falls	Medicare claims or MCBS survey responses
Fractures	Medicare claims or MCBS survey responses
Socioeconomic characteristics	MCBS survey responses
Overall health status	MCBS survey responses
CCI scores	Medicare claims

Abbreviations: ADL, activities of daily living; iADL, instrumental activities of daily living; MCBS, Medicare Current Beneficiary Survey.

Figure S1. Unadjusted Difficulties in Individual Activities

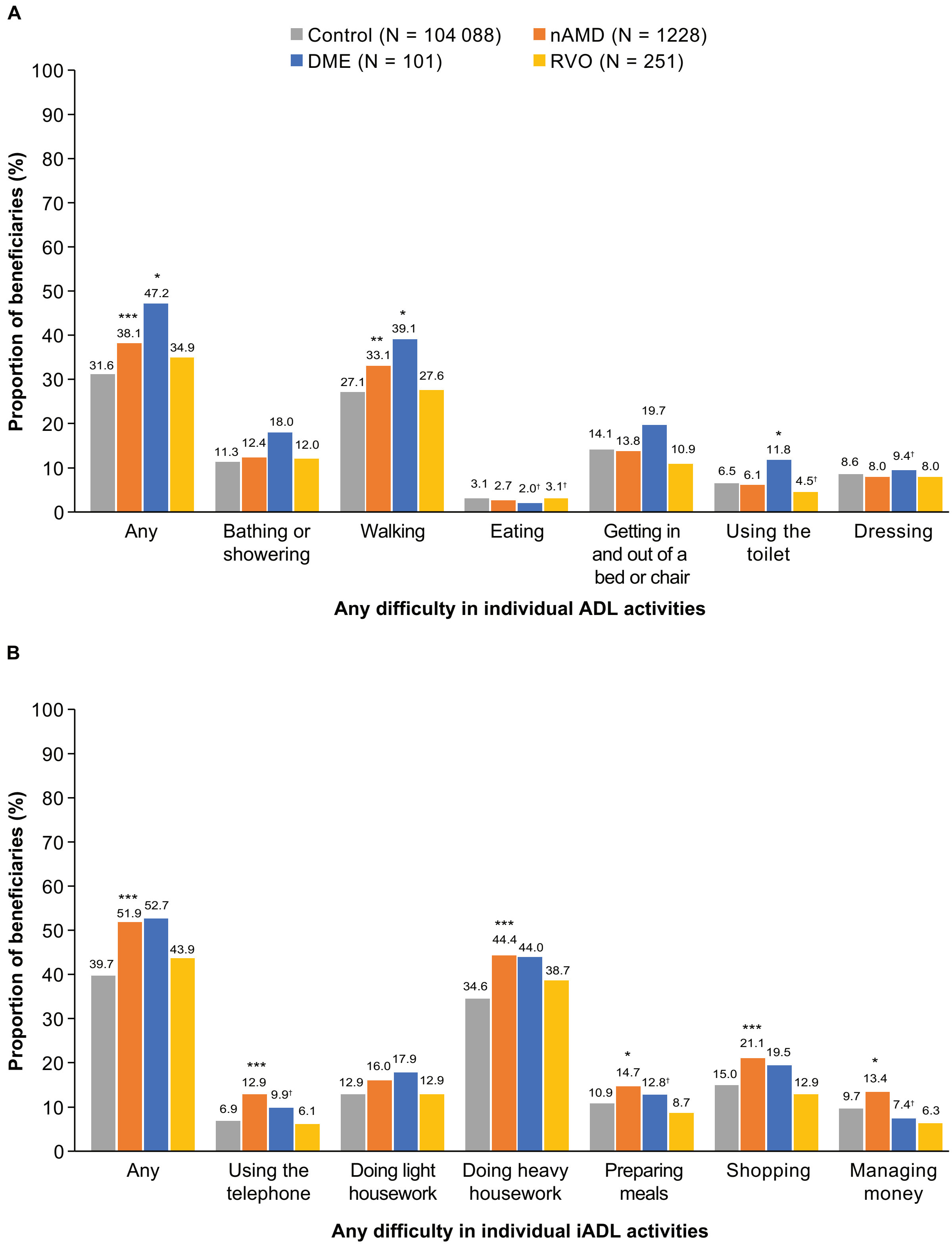

*$P<.05$; **$P<.001$; ***$P<.0001$ (vs control cohort).
†May not be reliable as estimate has a relative standardized error >30%.
